# The Role of Nondigestible Oligosaccharides in Alleviating Human Chronic Diseases by Regulating the Gut Microbiota: A Review

**DOI:** 10.3390/foods13132157

**Published:** 2024-07-08

**Authors:** Meiyu Yuan, Zhongwei Zhang, Tongying Liu, Hua Feng, Yuhuan Liu, Kai Chen

**Affiliations:** 1 State Key Laboratory of Food Science and Resource, Engineering Research Center for Biomass Conversion, Ministry of Education, Nanchang University, Nanchang 330047, China; yuanmeiyu9@163.com (M.Y.); redmaple9966@163.com (Z.Z.); 2 School of Public Health, Jiangxi Medical College, Nanchang University, Nanchang 330019, China; fenghua@ncu.edu.cn; 3 Jiangxi Maternel and Child Health Hospital, Nanchang 330108, China; wyyxliuty@163.com; 4 Chongqing Research Institute of Nanchang University, Chongqing 402660, China; 5 Shangrao Innovation Institute of Agricultural Technology, College of Life Science, Shangrao Normal University, Shangrao 334001, China

**Keywords:** nondigestible oligosaccharides, gut microbiota, short chain fatty acids, constipation, diabetes, obesity, depression, inflammatory bowel disease

## Abstract

The gut has been a focus of chronic disease research. The gut microbiota produces metabolites that act as signaling molecules and substrates, closely influencing host health. Nondigestible oligosaccharides (NDOs), as a common dietary fiber, play an important role in regulating the structure and function of the gut microbiota. Their mechanism of action is mainly attributed to providing a carbon source as specific probiotics, producing related metabolites, and regulating the gut microbial community. However, due to the selective utilization of oligosaccharides, some factors, such as the type and structure of oligosaccharides, have different impacts on the composition of microbial populations and the production of metabolites in the colon ecosystem. This review systematically describes the key factors influencing the selective utilization of oligosaccharides by microorganisms and elaborates how oligosaccharides affect the host’s immune system, inflammation levels, and energy metabolism by regulating microbial diversity and metabolic function, which in turn affects the onset and progress of chronic diseases, especially diabetes, obesity, depression, intestinal inflammatory diseases, and constipation. In this review, we re-examine the interaction mechanisms between the gut microbiota and its associated metabolites and diseases, and we explore new strategies for promoting human health and combating chronic diseases through dietary interventions.

## 1. Introduction

Chronic diseases refer to diseases that have a slow onset, slow progression, long course, and usually cannot be completely cured. In the past few years, the role of the gut microbiota in related diseases has been extensively studied, such as ulcerative colitis [1], irritable bowel syndrome [2], diabetes [3], Alzheimer’s disease [4], nonalcoholic fatty liver disease [5], and obesity [6]. In this case, the role of probiotics becomes particularly important. By regulating the balance of the gut microbiota, it is possible to maintain the stability of the gut environment, enhance intestinal barrier function, reduce gut inflammation levels, and help regulate energy metabolism and insulin sensitivity [7,8,9]. For example, in obese individuals, the diversity of the gut microbiota is decreased, which is usually accompanied by changes in key species [6,10,11,12,13] like *Firmicutes* and *Bacteroidetes* [14,15]. Diabetes mellitus type 2 (T2DM) is characterized by an impaired gut microbiota [16,17], and *Akkermansia* and *Enterobacter* have been found to have a causal role in the onset and progression of T2DM [18]. Nonalcoholic fatty liver disease (NAFLD) is regarded as a typical metabolic disorder at the intersection of obesity, metabolic syndrome, and T2DM [19], often with a proliferation of *Enterobacteriaceae* and *Escherichia coli* [20]. Dysbiosis of the gut microbiota has also been associated with inflammatory bowel disease (IBD), encompassing three main phenotypes—Crohn’s disease (CD), ulcerative colitis (UC), and IBD unclassified (IBDU) [21]. There are common microbial patterns among IBD patients, manifested at the phylum level as a loss of *Firmicutes* [22,23,24] and an increase in *Proteobacteria* and potential pathogenic bacteria [25,26,27,28]. At the genus level, *Clostridium* and *Escherichia coli* are significantly increased in IBD, whereas *Phascolarctobacterium*, *Faecalibacterium*, and *Roseburia* intestinalis are significantly decreased [27,29,30,31,32]. The gut–brain axis plays an important role in neurological diseases, including Alzheimer’s disease (AD), anxiety, and depression. The gut microbiota is linked to the central nervous system in a variety of ways, including regulation of immune responses, formation of metabolites (e.g., short chain fatty acids), and synthesis of neurotransmitters (e.g., serotonin and dopamine). Now some studies have shown that some microorganisms can regulate intestinal homeostasis and hinder the progression of neurological diseases [33,34,35,36,37,38,39,40,41]. In fact, diet is the main determinant that drives the growth of gut microbiota and regulates its interaction with the host. The lack of a sufficient functional microbiota and accessible carbohydrates (MAC) is the main reason for the disappearance of important species that regulate intestinal homeostasis [42,43]. Nondigestible oligosaccharides (NDOs) are a typical MAC that are easily broken down by the metabolic enzymes of *Bacteroides* and *Lactobacillus*. The use of oligosaccharide fermentation to improve intestinal health is a widespread and current focus [44,45]. The beneficial effects and promising prospects of NDOs on the host by targeting the gut microbiota and microbial metabolites are now generally accepted [46,47,48,49,50], including improvement of bowel movements, suppression of appetite, enhancement of postprandial glycemic response, and promotion of mineral absorption. This article describes the structure and sources of NDOs, as well as the factors affecting the microbial fermentation of NDOs, and then it elucidates the important role of oligosaccharides in chronic diseases by regulating the gut microbiota.

## 2. Classification and Sources of Oligosaccharides

Oligosaccharides are a class of carbohydrates that consist of 2 to 10 monosaccharide molecules, typically consisting of two or more different monosaccharides that are linked together in various ways through glycosidic bonds [51], classified as digestible or indigestible [52]. In some dietary oligosaccharides, the anomeric C atom (C1 or C2) of the monosaccharide unit has a configuration that makes their glycosidic bonds nondigestible to human digestive enzymes [52]. In addition to α-1,4 glycosidic bonds, the chemical bonds between monosaccharides in NDOs also contain α-1,6 glycosidic bonds or β-1,2 glycosidic bonds that cannot be degraded by human digestive enzymes. Nondigestible oligosaccharides Oligosaccharides belong to prebiotics along with fiber polysaccharides, which are selectively fermented and cause specific changes in the composition and/or activity of the gastrointestinal microbiota, thereby benefiting the wellbeing and health of the host [53,54]. The source and structural characteristics of the main functional oligosaccharides are listed in Table 1.

## 3. The Impact of Different NDOs on the Diversity of the Gut Microbiota

Nondigestible oligosaccharides (NDOs) are typically fermented by microorganisms via enzymes related to carbohydrate metabolism [64,65]. For example, LAB can degrade FOS and GOS by encoding β-FFase and β-galactosidase [66]. Different NDOs often have many differences, such as the source, degree of polymerization, glycosidic bonds, and overall structural complexity (e.g., side chains) [67,68,69]. These differences result in a diverse microbiota for fermenting NDOs, manifested by varying degrees of beneficial bacterial proliferation, inhibition of harmful bacteria, and differences in acid and gas production [70].

### 3.1. Degree of Polymerization (DP)

In fact, NDOs with different DPs can be utilized by different strains. For example, DP3 oligofructose (FOS) promoted the growth of *Bifidobacterium* species in vitro while inhibiting the proliferation of *Clostridia*, and it showed superior selective stimulatory activity compared to other FOS [71]. In previous work, Gopal et al. [72] demonstrated that *Bifidobacterium lactis DR10* consume oligosaccharides (GOS) with higher DP, while *Lactobacillus rhamnosus DR20* prefer to use galactose disaccharides. Zhao et al. [73] evaluated the prebiotic ability of bamboo shoot shell xylo-oligosaccharides (XOS) with different DPs, and showed that XOS with a high DP (>X5) led to an increase in the abundance of beneficial bacteria, such as *Phascolarctobacterium* and *Bacteroides*, while low-DP XOS with more X2–X3 components promoted the proliferation of other beneficial bacteria, including *Lachnochlostridium*. Immerzeel et al. [74] and Falck et al. [75] demonstrated that *Bifidobacterium* adolescentis and *Lactobacillus acidophilus* predominantly utilized the X2–X3 fractions.

Degradation of high-DP NDOs by microorganisms usually requires more energy consumption because microorganisms preferentially degrade high-DP NDOs to low-DP NDOs, which limits the metabolic capacity of the gut microbiota [76]. On the contrary, the decomposition of low-DP NDOs is relatively simple and requires less energy by microorganisms, thus it has a stronger ability to support the growth and proliferation of the gut microbiota [77]. For example, another study on oligosaccharides showed that the component with DP 3–4 (≥80%) was utilized by all probiotic strains, the portion with DP 5–6 was utilized by some strains, and the portion with DP ≥ 7 was only utilized by *Lactic acid bacteria (LAB)* and *Bifidobacteria* [78]. Similarly, isomaltose from DP 3–5 was more indigestible than DP 2, and although it had a higher chance of reaching the colon, its fermentation selectivity for beneficial probiotic species was lower [79,80].

The impacts of NDOs with different DPs on the major metabolites produced by the gut microbiota differ. Firstly, NDOs with different DPs can lead to the generation of different types and proportions of short chain fatty acids (SCFAs) by the gut microbiota. The fermentation of NDOs with high DP is slower, gradually releasing more butyric acid, and can serve as a potential substrate to induce the proliferation of butyric acid-producing bacteria. Low-DP NDOs are rapidly fermented, producing more acetic acid and propionic acid [73,81]. Different amounts of gas products, such as hydrogen and carbon dioxide, are also generated during the fermentation of NDOs with different DPs. Usually, NDOs with high DP have less gas production due to slower fermentation rate, while NDOs with low DP have a faster fermentation rate and more gas production.

### 3.2. Glycosidic Bond

The fermentation process of NDOs in the intestine involves the ingestion of oligosaccharides that are first recognized by modular glycanase on bacterial membranes and then hydrolyzed into oligosaccharides with low DP. Afterward, the low-DP NDOs are transported into the cell through membrane protein transporters and broken down by enzymes into disaccharides and monosaccharides [82]. For example, GOS is transported to cells through galactoside-pentose-hexuronide permease (LacS) and hydrolyzed into galactose and glucose by β-galactosidase, which is then directed to the Leloir pathway and glycolysis [83]. FOS is first taken up by *L. acidophilus* cells through ABC transporters and degraded into β-glucoside, which is further decomposed into fructose and glucose by fructosidase and β-furanosidase, respectively [45]. In subspecies of *Bifidobacterium infantile*, HMO substrates are transported intact through ABC transporters, and subsequent hydrolysis is mediated by various cytoplasmic glycosidases, including alpha-fucosylase and 2,3/6-α sialidase [45,82].

The different structures of NDOs have different effects on the gut microbiome, including glycosidic bond type and connection mode. For example, α-(1→3)- and α-(1→2)-linked glucose oligosaccharides (GlcOSs) formed during isomaltooligosaccharide (IMO) production promote the growth of *Bifidobacterium* spp. and *Lactobacillus* spp. [84], and their fermentation selectivity (against probiotic species in gut bacteria) is higher than that of only α-(1→6)-linked IMOs [85]. Zeng et al. [86] studied the effect of NDOs with different disaccharide bonds on selected *Lactobacillus*, and most of the species had low growth rates on IMOs with α-(1→6) except *L. fermentum FUA 3589*, where β-(1→4)-linked fibrous disaccharides and β-(1→6)-linked gentian disaccharides were able to significantly promote the proliferation of *L. brevis ATCC 8287*, *L. rhamnosus ATCC 53103*, *L. plantarum WCFS1*, and *L. gasseri ATCC 33323*. Similarly, Sanz et al. [70] investigated the prebiotic index (PI) of disaccharides, which was higher for α-glucose disaccharides than for β-types, except for α, α-alginose. Djouzi Z. et al. [87] compared the effects of β-oligofructose, β-oligogalactose, and α-oligosaccharides (non-disaccharides) on the metabolism of the gut microbiota, and compared to α-oligosaccharides, oligofructose and oligogalactose containing β-glycosidic bonds were the preferred substrates for the growth of *Bifidobacteria*, having a great impact on microbial composition, which has been found in several studies [88,89]. More importantly, it has been reported that the intake of GOS containing β-1,3, β-1,4, and β-1,6 glycosidic bonds showed better prebiotic effects in healthy volunteers [90]. In addition, Kittibunchakul et al. [91] concluded that the fermentation activity of *Bifidobacterium* and *Lactobacillus* as well as *E. faecium* on GOS mainly containing β-1,3 and β-1,6 glycosidic bonds was better than that on GOS containing a single β-1,4 glycosidic bond. This was similar to the conclusion of Cardelle Cobas [92], who found that *Bifidobacteria*, *LAB*, and *Streptococcus* preferred galactose containing β-1,6 glycosidic bonds, but not β-1,4 glycosidic bonds. In conclusion, the growth-inducing ability of probiotics is related to the type of glycosidic bond. Therefore, it is important to understand the structure–activity relationship of NDOs.

### 3.3. Linear and Branching Structures

The number of substituents in NDOs will affect the microbial fermentation rate. Low-substituted NDOs are preferentially fermented, leading to the accumulation of relatively high-substituted NDOs, indicating that the presence of substituents delays or completely hinders fermentation [93]. The number of substituents in NDOs will affect the microbial composition. It has been reported that the presence of substituents on XOS can affect *Bifidobacterium* fermentation, resulting in different amounts of lactic acid. Kabel observed that the lactic acid content in unsubstituted XOS was higher than that in substituted XOS [94]. That is, unsubstituted XOS was fermented by more gut microorganisms. In addition, previous studies have described the preference of bifidobacteria for low-substitution XOS fermentation in vitro and in vivo. This indicates that microorganisms may preferentially grow or be inhibited based on the structure of NDOs, altering the composition of the entire microbial community during the fermentation process. Similar observations were described by Englyst et al. [95] for the fermentation of arabinose side chains of pectin, xylan, and arabinogalactan by mixed populations of human fecal bacteria. These results might point to the suggestion that the number of substituents present per oligomer influences the rate of fermentation.

The type of substituents in NDOs will affect the microbial fermentation rate and composition. When fermented in vitro with human feces, the fermentation speeds of linear XOS and arabinose-substituted XOS (AXOS) were faster than that of acetylated XOS (AXOS), and XOS containing the 4-O-methylglucuronic acid group (GlcAmeXOS) was the slowest [96]. On the other hand, *Bacteroidetes* showed superior growth performance when XOS was used as the sole carbon source compared to AXOS [97]. The process of XOS degradation by *Bacteroidetes* involves extracellular degradation and debranching of the skeleton, followed by intracellular hydrolysis of the oligomers, which ultimately produces acetate, propionate, and butyrate as the major metabolic end products [98]. It has been proven that microbial degrading enzymes do not easily contact NDOs with a high branch content, resulting in a reduction in the microbial fermentation rate [99].

In addition, the chain length and shape of NDOs are equally important. For example, long-chain IMO has stronger resistance to hydrolysis and degradation in the upper intestine and is more effective than short-chain IMO in stimulating the growth of *Bifidobacterium*, *Prevotella*, and *Lactobacillus*. *Bifidobacteria* have selective specificity in utilizing FOS with different chain lengths. Generally, short-chain FOS are preferentially fermented, followed by long-chain FOS [100]. Cyclic IMO has been shown to inhibit *Streptococcus* [80,101,102,103]. Wheat arabinoxylan hydrolysates with more branches can only (partially) be fermented by *Bifidobacterium* and *Bacteroidetes*.

From the above viewpoints, the degree of polymerization, substituents, and glycosidic bond type have significant impacts on the fermentation activity of probiotics. However, the reasons for the preference of the microbiota for oligosaccharides lack in-depth investigation, and the metabolic mechanisms of microorganisms for oligosaccharides with different structures need further study.

## 4. The Impact of Different NDOs on the Diversity of the Gut Microbiota

### 4.1. T2DM

Diabetes is a chronic disease characterized by hyperglycemia, caused by absolute or relative insufficiency of insulin secretion and impaired utilization. At present, increasing dietary fiber intake is one of the recommendations for patients with T2DM [104]. In recent years, many studies have shown that, including in vivo and in vitro experiments, NDOs can effectively improve diabetes by regulating the gut microbiota to enrich or decrease specific microorganisms. For example, in T2DM rats, the abundance of *Firmicutes* increased at the phylum level, while the abundance of Bacteroidetes, Actinobacteria, and *Verrucomicrobia* decreased [105]. At the genus level, *Bifidobacterium*, *Roseburia*, *Faecalibacterium*, *Bacteroides*, and *Akkermansia* abundance decreased, while that of *Desulfovibrio*, *Oscillibacter*, *Fusobacterium*, *Ruminococcus*, and *Blautia* increased [106,107]. Weninger et al. [107] showed that FOS reduced species α-diversity after 16 weeks of treatment in T2DM model rats, that the relative abundance of *Bifidobacterium* increased, and the relative abundance of Ruminococcus decreased significantly. This result was also found in the later study of FOS and GOS [108], with the difference that *Phascolarctobacterium*, *Coprococcus,* and *Oscillospira* abundance was also decreased.

In addition, NDOs can regulate insulin sensitivity, blood glucose, and lipid metabolism by influencing microorganisms to produce beneficial metabolites, such as SCFAs, amino acid metabolites, and bioactive polypeptides, thereby alleviating the onset and progression of diabetes.

**Figure 1:**
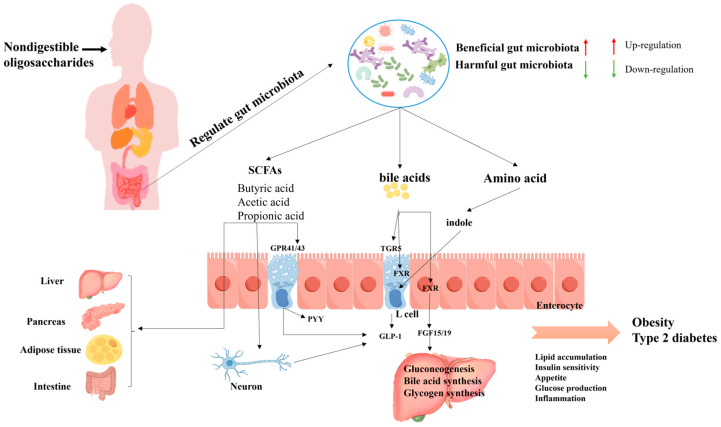
The effect of NDOs on T2DM and obesity by regulating gut microbiota composition and metabolites. (1) SCFAs: SCFAs can promote the release of PYY and GLP-1 from intestinal endocrine L cells, increase energy consumption of adipose tissue, and reduce lipid accumulation and inflammation. In the liver, SCFAs affect the generation of fat and glucose. In the pancreas, SCFAs promote insulin secretion. SCFAs can transmit neural signals to maintain homeostasis of systemic functions. (2) Amino acids: amino acids are converted by intestinal bacteria into other bioactive metabolites, such as indole, which can stimulate L cells to secrete GLP-1, thereby promoting insulin secretion and inhibiting glucagon secretion. (3) Bile acids (BAs): BAs are converted to secondary bile acids through uncoupling, dehydroxylation, and epimerization by intestinal bacteria. BAs act on two receptors (FXR and TGR5) to regulate glucose homeostasis. In addition, the activation of FXR by BAs promotes the release of FGF15/19, which promotes hepatic glycogen synthesis and reduces BA synthesis and gluconeogenesis in the liver.

(1) SCFAs are currently the most studied metabolites. According to reports, SCFAs (acetate, propionate, and butyrate) can reduce body weight and hepatic steatosis in mice and reverse high-fat diet (HFD)-induced metabolic abnormalities by reducing the expression of PPAR-γ in the liver and adipose tissue [109]. Similarly, acetate can inhibit fat production in the liver and reduce lipid accumulation in adipose tissue [110]. SCFAs regulate pancreatic insulin secretion, prevent insulin resistance, and increase insulin sensitivity. Propionate has shown important biological functions in in vitro and in vivo studies. It enhances glucose-stimulated insulin release and maintains the function and numbers pancreatic β-cells through several mechanisms, including inhibition of apoptosis, promotion of cell proliferation, and reduction of reverse differentiation of α-cells to β-cells [109]. Therefore, propionate helps to maintain the integrity and stability of pancreatic islet function. In addition, it has been reported that butyrate can improve insulin sensitivity and prevent insulin resistance in mouse models and large population cohorts. Its mechanism of action is related to promoting energy expenditure and stimulating mitochondrial function [111,112]. In addition, SCFAs can inhibit appetite and energy intake by promoting the production of satiety hormones. SCFAs (acetate, propionate, and butyrate) act as signaling molecules to maintain host energy homeostasis by activating G protein-coupled receptors GPR41 and GPR43 to regulate insulin levels and inhibit hepatic gluconeogenesis [113]. It also promotes the secretion of glucagon-like peptide-1 (GLP-1) and peptide YY (PYY). GLP-1 participates in glucose homeostasis primarily by reducing blood glucose levels and improving insulin secretion and resistance. PYY, as a hormone mediating satiety, is secreted from endocrine L cells in the distal intestine after the body intakes enough food. SCFAs play a crucial role in the development of T2DM and can be used as markers of metabolic disorders or homeostasis [114].

(2) Amino acids and their metabolites: the intake of NDOs can produce a variety of amino acid metabolites, which have an impact on blood glucose levels and insulin sensitivity. Some amino acids can be converted into various bioactive metabolites by specific gut microorganisms, such as indole, imidazole propionate, and tyramine. For example, tryptophan (Trp) can be metabolized into indole by various bacteria and can regulate the secretion of GLP-1 [109]. Trp can also be converted into indolepropionic acid (IPA) by some intestinal microorganisms, such as *C. caloritolerans*, and *C. paraputrificum*. IPA can significantly reduce fasting blood glucose and insulin levels in rats, as well as homeostasis model assessment of insulin resistance (HOMA-IR), improving insulin resistance [115]. It has been reported that fecal metabolic profiling showed that the content of glycogen amino acids, such as proline, serine, and leucine, increased after NDO intervention in T2DM model rats [116]. This result was also confirmed by Chen et al. [117], who found that increases in serine and glutamine could reduce the risk of T2DM progression. In conclusion, NDOs can influence glucose metabolism (especially glucose metabolism), lipid metabolism, and protein and carbohydrate metabolism by regulating the gut microbiota and fecal metabolites [116,117]. On the other hand, hundreds of metabolites of the gut microbiota can be fed back to the brain, which plays a key role in influencing many gastrointestinal processes through the autonomic nervous system and the hypothalamus–pituitary–adrenal axis [118] (Figure 1).

### 4.2. Obesity

Several studies have revealed the role of the gut microbiota in host physiology, behavior, and metabolism, providing new insights into the pathogenic mechanism of obesity.

Firstly, gut microbial dysbiosis is a common feature in obese patients and obese animal models. Studies have shown that prebiotic oligosaccharides, such as FOS, XOS, GOS, and COS, have an anti-obesity effect by regulating the gut microbiota [49]. Generally, obese individuals have higher proportions of *Firmicutes* and *Bacteroidetes*. A study on an obese mouse model showed that the administration of NDOs increased the abundance of *Bacteroidetes*, decreased the abundance of *Firmicutes*, and changed more than 100 bacterial taxa [119]. Long et al. [120] demonstrated that the XOS diet reduced visceral fat in mice, which was associated with changes in the gut microbiota. Specifically, they observed a significant phylum-wide shift of increased *Bacteroidetes* and decreased *Firmicutes*. At family and genus levels, a significant increase in f_S24-7_Unclassified and decreases in *Streptococcaceae*, *02d06*, *Coprococcu*, *Ruminococcus*, and *Lactococcus* were observed. In addition, the increase in Proteobacteria containing Gram-negative lipopolysaccharide (LPS) is also a microbial signature of intestinal dysbacteriosis in mice fed a high-fat diet (HFD) [121]. HFD leads to a sustained increase in plasma LPS levels (i.e., metabolic endotoxemia) as well as a systemic inflammatory response, which play a promoting role in the development of insulin resistance in obese patients. In a study of obese women, *Bifidobacterium* and *Faecalibacterium prausnitzii* abundance was increased after intervention with FOS, and it was proven that the changes in the gut microbiota were related to the changes in fat mass, serum LPS level, and metabolism (hippuric acid, lactate, and plasma protein C) [122]. Similarly, Thiennimitr et al. [121] demonstrated that consumption of XOS reduced metabolic endotoxemia in obese mice. The results indicated that NDOs could inhibit the growth of pro-inflammatory bacteria, such as certain species within the *Bacteroidetes* and *Firmicutes* phyla (e.g., *Ruminococcus*, *Proteobacteria*, and some toxin-producing *Clostridium* spp.), and promote the growth of beneficial bacteria, such as *Bifidobacterium*, *Lactobacillus*, and *Prevotella*, thereby helping to alleviate intestinal inflammation and reduce the risk of obesity. In summary, manipulation of the composition of the gut microbiota through NDOs has been considered as a possible approach for the prevention and treatment of obesity.

The direct cause of obesity is that the rate of fat synthesis exceeds the rate of fat consumption. Therefore, blocking fat synthesis and increasing energy consumption is the most effective strategy to combat obesity. The impact of NDOs on weight loss is mainly attributed to the production of SCFAs by the gut microbiota [123], as they play an important role in regulating food intake and energy metabolism [124]. Acyl CoA carboxylase (ACCase) is a key enzyme in the process of fatty acid synthesis. By inhibiting ACCase activity, the synthesis of fatty acids can be reduced, which helps to prevent obesity. Acetate is catalyzed to acetyl-CoA and butyrate is converted to butyryl-COA as an inhibitor of fatty acid synthesis of rate-limiting enzyme (ACCase) to prevent obesity [125]. Butyrate is enriched by the intake of oligosaccharides, and regulating the ratio of butyrate to acetate in the gut microbiota is a potential strategy for the treatment of obesity. Research has shown that GOS improves lipid metabolism in mice by regulating the synthesis of SCFAs [126]. Dietary supplementation of MOS can inhibit appetite and systemic insulin resistance in obese mice by remodeling gut microbial composition and enhancing the formation of SCFAs [127]. Chen et al. [125] found that XOS changed the gut microbiota and affected the levels of SCFAs. After removing the gut microbiota, SCFA levels were significantly decreased, indicating that XOS indirectly affected the generation of SCFAs by regulating the metabolic activities of the gut microbiota.

NDOs can regulate the synthesis and release of bile acids by affecting the gut microbiota associated with bile acid (BA) metabolism, such as *Clostridia*, *Bacteroidetes*, *Bacteroides*, *Lactobacillus*, and *Bifidobacterium* [49]. The G protein-coupled bile acid receptor (TGR5) is one of the key receptors for BA. Under the action of *Clostridia*, bile acids can be converted to deoxycholic acid (DCA) and lithocholic acid (LCA), which activate TGR5 and enhance the cyclic adenosine monophosphate/protein kinase A (cAMP-PKA) signaling pathway. Activation of TGR5 also promotes conversion of glucagon to GLP-1. Lun et al. [128] revealed the dynamic balance relationship between the gut microbiota and bile acids.

The study found that after COS intervention, the gut microbiota can promote the release of bile acids and then activate TGR5, enhancing brown fat thermogenesis and the TGR5-dominated fatty acid oxidation signaling pathway, thus playing a role in weight loss and lipid reduction. Farnesoid X receptor (FXR) is another key receptor for BA, and several studies have shown that activation of FXR promoted adipose tissue browning and reduced obesity in mice [129]. Mechanistically, activation of FXR induces the release of fibroblast growth factor 15 (FGF15) or FGF19, which reach hepatocytes through the portal vein, thereby inhibiting BA synthesis in the liver [130]. In addition, FGF19 promotes hepatic glycogen synthesis and inhibits hepatic gluconeogenesis. Other studies have shown that GOS significantly reduced the concentration of bile acids in the small intestine, improved glucose metabolism, and influenced lipid absorption by regulating lipid digestion [131,132]. Pectin oligosaccharides have also been shown to mediate cholesterol metabolism through the gut microbiota and their metabolites [133].

The gut microbiota can utilize NDOs to generate nutritional metabolites that act as “messengers” for the bacterial community, influencing host energy homeostasis and regulating host metabolism, thus contributing to the fight against obesity (Figure 1).

### 4.3. Depression

NDOs may influence the development of depression by regulating the gut microbiota. For example, FOS inhibits depression-associated bacteria such as *Anaerostipes*, *Lachnospiraceae* incertae sedis, *Oscillibacter*, *Proteobacteria*, and *Streptococcus*, while effectively promoting the growth of beneficial bacteria with antidepressant properties, such as *Cyanobacteria*, *Lachnospiraceae*, *Oxalobacter*, *Desulfonema* spp., *Peptoclostridium*, and *Parabacteroides* [134]. This was consistent with the study by Burokas et al. on the anti-anxiety properties of FOS [135]. Among them, *Cyanobacteria* are considered beneficial and are known for producing pharmacologically important metabolites with antidepressant properties. *Desulfonema* spp. produce H2S as a bacterial energy source. A high level of H2S has shown antidepressive effects in an animal model. The abundance of *Oxalobacter* increased in rats treated with anti-anxiety drugs [134].

In addition, the combination of FOS and GOS has been shown to reduce depressive symptoms [136]. Specifically, the combination of FOS and GOS increased levels of hippocampal brain-derived neurotrophic factor (BDNF) and cortical serotonin. At the same time, it reduced elevated corticosterone levels and body temperature. Three weeks of chronic social stress significantly reduced social interaction, whereas FOS+GOS administration protected against this effect. The combination of FOS and GOS also reduced anxiety levels as measured by the open space test and the maze test. At the microbial level, there is evidence that the combination of the two can prevent the change in the ratio of *Actinomycetes* and *Proteobacteria*, which is a typical feature of patients with major depression [137]. Chi et al. [134] conducted a comparative analysis of FOS and the standard antidepressant fluoxetine, noting that they had similar effects and that both FOS and fluoxetine promoted the growth of *Dialister* spp. A recent cohort study reported that *Dialister* was not found in depressed patients [138]. In other words, FOS and fluoxetine may improve depressive symptoms by regulating the presence of *Dialister*. In addition, *Eubacterium* [139], *Lactobacillus* [139,140], and *Oxalobacter* [141] are also considered beneficial. Overall, NDOs show antidepressant activity, and part of this effect is achieved by regulating the functional gut microbiota, although the exact mechanism in depressed patients requires further investigation.

The microbiota–gut–brain axis (MGBA) has been considered as a potential pathway for the treatment of neurodegenerative diseases [142] involving the autonomic nervous system (ANS), the enteric nervous system (ENS), and the hypothalamic–pituitary–adrenal axis (HPA). Gut microbes can affect the brain through a variety of pathways, including neural, immune, and endocrine pathways, and microbial metabolites, and even stimulate the vagal nerve endings of the gut through neurotransmitters produced by microbes themselves [143]. Neurotransmitters known to be affected by the gut microbiota include SCFAs [144], γ-aminobutyric acid (GABA) [145], Trp [146], and serotonin (5-HT) [147]. For example, valeric acid can alleviate intestinal injury, protect neurons, and regulate neurotransmitters [148]. And the increase in butyrate may directly affect the central nervous system [149,150,151]. This view was validated in a pig model, where oral butyrate was found to affect brain metabolism and hippocampal neurogenesis [152]. Therefore, NDO supplementation may inhibit the development of depression by enhancing the formation of SCFAs, including improving cognitive functioning and alleviating anxiety behaviors. Another important pathway is tryptophan → 5-hydroxytryptophan → serotonin. The gut microbiota plays an indispensable role in intestinal tryptophan metabolism. Tryptophan is the only precursor for the synthesis of the neurotransmitter 5-HT, which influences the development of depression [153]. Kelly et al. [154] found that transplantation of feces from depressed patients into recipient animals resulted in these recipient animals showing behavioral and physiological characteristics of depression, and the metabolic pathway of tryptophan was changed. Deng et al. [155] demonstrated that feruloylated oligosaccharides (FOs) exhibited antidepressant and anxiolytic effects by regulating 5-HTP, suggesting that the gut microbiota and microbial metabolism were key mediators supporting protection of the nervous system by FOs. Similarly, Zhang et al. [156] revealed that Morinda officinalis oligosaccharide (MOO) regulated the 5-HT synthesis pathway (Trp → 5-HTP → 5-HT) in the gut microbiota, and the elevated 5-HTP from the gut microbiota was absorbed into the blood and then crossed the blood–brain barrier to improve the 5-HT level in the brain. These findings suggest that the gut microbiota can regulate neurotransmitter levels to affect the development of depression.

**Figure 2:**
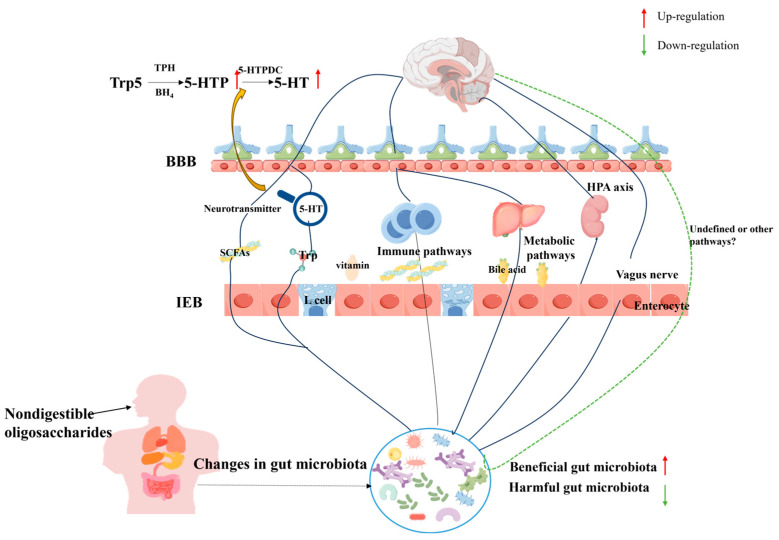
The gut microbiota and the brain communicate in both directions through the immune system, intestinal endocrine signals, neurotransmitters, amino acids, BAs, SCFAs, the hypothalamic–pituitary–adrenal axis, etc.

Gut microbial dysbiosis may lead to physiological and behavioral disorders by inducing neuroinflammation in the central nervous system. NDOs alter neural function by manipulating the gut microbiota. Currently, the exact mechanisms by which NDOs play a role in depression are unknown, and it is difficult to definitively show how many or which key pathways are responsible for the pathophysiology of depression. However, it is still a very important research direction to regulate the gut microbiota and its metabolites to affect host metabolic pathways, thereby alleviating depressive symptoms (Figure 2).

### 4.4. Inflammatory Bowel Disease (IBD)

**Figure 3:**
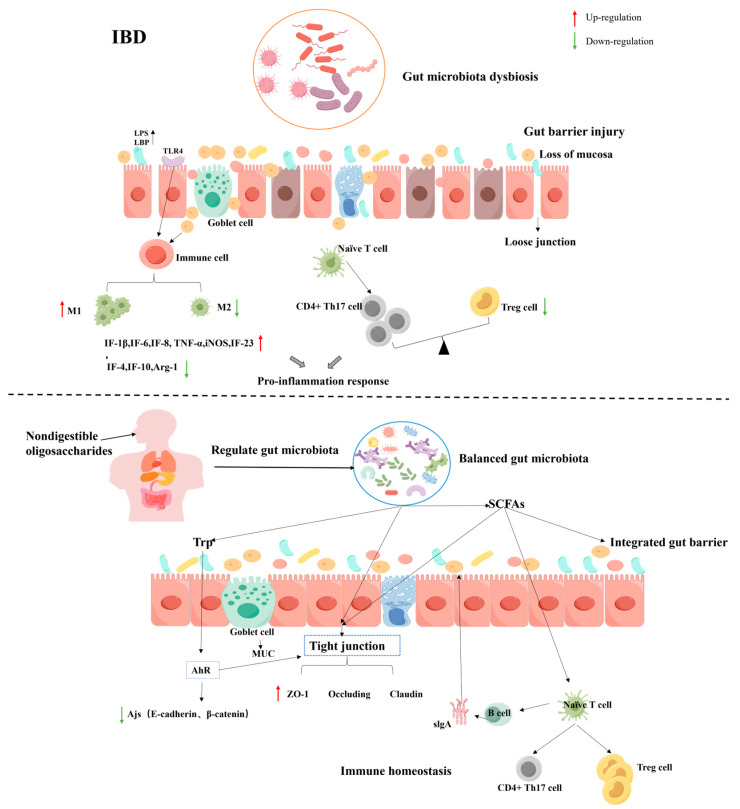
NDOs alleviate IBD progression by regulating the gut microbiota. (1) NDOs promote the proliferation of beneficial bacteria and correct intestinal dysbiosis in IBD patients. (2) SCFAs can improve the barrier function and reduce the permeability of the intestinal barrier by affecting the maturation of intestinal epithelial cells (IECs) and regulating the expression of tight junction proteins (claudin1, occludin, and ZO-1). SCFAs also affect the interaction between immune cells (T cells, B cells), regulate the expression of pro-inflammatory/anti-inflammatory cytokines, and reduce intestinal inflammation. (3) Microbial metabolites of Trp regulate the expression of adhesion junction proteins (E-cadherin and β-catenin) and maintain the integrity of the intestinal barrier.

Inflammatory bowel disease (IBD) is a common non-specific chronic gastrointestinal inflammatory disease, including Crohn’s disease (CD) and ulcerative colitis (UC). The mechanisms by which oligosaccharides alleviate IBD by regulating the gut microbiota mainly include: (1) enriching the diversity of the gut microbiota; (2) protecting the intestinal barrier; and (3) regulating T-cell differentiation and related signaling pathways (Figure 3). Recent studies have verified that breast milk oligosaccharides (HMOs) alleviated sodium disulfide (DSS)-induced ulcerative colitis by improving intestinal barrier function and regulating the gut microbiota [48]. The gut microbiota of colitis model mice treated with unsaturated alginate oligosaccharides (UAOS) was altered, with increased abundance of *Firmicutes* and *Actinomycetes* and decreased abundance of *Bacteroidetes*, and the results showed that the protective effect of UAOS treatment was attributed to the maintenance of mucosal barrier function and suppression of immune injury by regulating the gut microbiota [157].

Increasing evidence shows that functional oligosaccharides, as natural active substances, can effectively improve IBD [100,158].

(1) NDOs can alleviate intestinal inflammation in animals by regulating the gut microbiota. Gut microbial dysbiosis leads to ecological imbalance of the intestinal mucosa, increased production of pro-inflammatory cytokines, and reduced the production of anti-inflammatory cytokines. It was found that the levels of *Escherichia coli* and *Enterococcus faecalis* in the feces of patients with IBD were significantly higher than those of normal people, while the abundance of *Lactobacillus* and *Bifidobacterium* was significantly lower [159]. In addition, FOS alleviated intestinal inflammation by promoting the growth of *LAB* [157]. SCFAs can be absorbed by intestinal epithelial cells (IECs) and inhibit the production of pro-inflammatory cytokines IL-8 and IL-6, thereby alleviating the inflammatory response of colonic epithelial cells. In conclusion, NDOs can alleviate the symptoms of IBD by reducing the abundance of pro-inflammatory bacteria, increasing the abundance of anti-inflammatory bacteria, and promoting the production of SCFAs [160].

(2) Protecting the intestinal barrier. In animal models of IBD, alterations in tight junction proteins (e.g., claudin1, occludin, and ZO-1) and adhesion junction proteins (e.g., E-cadherin and β-catenin) are usually accompanied by increased intestinal permeability and disruption of intestinal structure. In fact, it helps to restore the integrity of the mucosal barrier by regulating gut microbiota diversity [161]. For example, HMOs restored the expression of tight junction proteins and MUC-2, inhibited the production of LPS and LBP, and restored the integrity of the intestinal barrier in colitis model mice [48]. SCFAs may provide energy for IEC, promote epithelial cell proliferation and differentiation, and enhance intestinal barrier function, which may be related to their regulation of the AP-1 signaling pathway [162]. In addition, konjac oligosaccharides increased the concentration of SCFAs in the mice colon, which could promote IL-18 and repair the integrity of IECs [163]. Essential aromatic amino acids (e.g., Trp) can be metabolized into indoles and their derivatives, and these metabolites can activate the aryl hydrocarbon receptor (AhR), which is closely associated with the pathogenesis of IBD [164]. This is consistent with the findings of Xia et al., who found that three microbial metabolites of Trp alleviated IBD by improving tight junctions [165].

(3) Regulation of immune homeostasis. Toll-like receptors (TLRs) in intestinal epithelial cells and immune cells recognize and respond to different microbial structural motifs. When pathogens invade, macrophages recognize pathogen-associated molecular patterns (PAMPs) through TLRs and secrete various proinflammatory cytokines (such as TNF-α, IL-1 β, IL-6, IL-12, etc.). TLR-4 plays a crucial role as a receptor for LPS in the pathogenesis of UC. The number of Treg cells is decreased in patients with IBD. SCFAs can induce the differentiation of T cells into Treg cells, promote the synthesis of anti-inflammatory cytokines to regulate the immune response, and restore the balance of Th1/Th2 and Th17/Treg cells. A study showed that XOS was metabolized into SCFAs, and propionate and butyrate can promote the differentiation of CD4+ T cells into Treg cells, thereby slowing down inflammatory bowel disease [166]. Similarly, feruloylated oligosaccharides (FOs) promoted the percentage of Treg cells and the production of corresponding specific cytokines, thereby regulating the immune homeostasis of Th17/Treg cell in IBD model mice [167]. In addition, the gut microbiota can also stimulate B cells (Breg) to produce inhibitory cytokines (e.g., IL-10 and TGF-β) that affect other immune cells and immunoglobulins (e.g., sIgA). Nowadays, numerous studies have confirmed the effectiveness of oligosaccharides in improving IBD by modulating the gut microbiota, both in animal models and in human experiments [168].

### 4.5. Constipation

Intestinal motility is regulated by the interaction of the intestinal immune system, intestinal secretions, the intestinal microbiota, and their fermentation products, and impaired intestinal motility can lead to constipation [169]. The increase in the abundance of beneficial bacteria such as *Bifidobacterium* and *Alistipes*, as well as the decrease in the abundance of colonic transport-associated bacteria (*Oscillospira* and *Odoribacter*), contributes to the relief of constipation [170]. NDOs, such as FOS, IMO, and GOS, can significantly improve defecation frequency and shorten colonic transit time, with few side effects [171]. In addition to the fact that NDOs themselves occupy intestinal volume to increase fecal volume and promote intestinal motility, several studies have shown that NDOs also have significant benefits in alleviating constipation in terms of microbiota remodeling [172,173,174,175]. Specifically, *LAB* utilize FOS to improve fecal consistency and reduce defecation time [176]. IMO effectively improves fecal consistency and frequency of defecation of subjects, which may be attributed to increased abundance of some microorganisms after colonic fermentation [174,175]. Lotus seed oligosaccharides alleviate constipation by stimulating the growth of beneficial bacteria in the host intestine to promote intestinal motility and defecation [177]. In addition, Zhang et al. demonstrated that COS could improve constipation by regulating the production of intestinal metabolites. This regulatory effect was mainly achieved by reshaping the structure of the gut microbiota in constipated mice [178].

Intake of NDOs increases the amount of substrate for probiotic fermentation and promotes the production of SCFAs. SCFAs, particularly butyrate, acetate, and propionate, are related to stimulation of the growth of colonic epithelial cells, thereby restoring intestinal morphology and increasing intestinal motility to alleviate constipation [179]. Current research suggests that butyrate improves intestinal motility by affecting water and electrolyte metabolism. Similarly, COS relieves constipation by restoring deteriorated water–electrolyte metabolism, including AQP3/4 and ENaC-β/γ expression. Among them, AQP3/4 transports water from the intestinal lumen to the colonic epithelium and ENaC-β/γ mediates the uptake of Na^+^ into colonic epithelial cells [178,180].

In the current literature, the improvement of constipation by NDOs may be related to the regulation of some metabolic pathways, including lipid metabolism (e.g., sphingolipid metabolism, glycerophospholipid metabolism, and arachidonic acid metabolism), bile acid metabolism, and tryptophan metabolism [169,180]. Sphingolipid metabolism and glycerophospholipid metabolism have been proven to be associated with inflammatory responses in intestinal disorders. Inflammation damages the integrity of the intestinal epithelium, which reduces the secretion of neurotransmitters and hormones, affects gastrointestinal motility, and induces constipation [181]. Previous studies have confirmed that gastrointestinal hormones play roles as neurotransmitters and neuromodulators in the central and peripheral nervous systems [182]. They promote intestinal peristalsis and transportation of contents. Endothelin (ET), somatostatin (SS), and vasoactive intestinal peptide (VIP) are inhibitory peptide neurotransmitters, while melatonin (MLT) and substance P (SP) are excitatory peptide neurotransmitters. Acetylcholinesterase (AchE) is the primary stimulant of spontaneous contractions in colonic smooth muscle. Therefore, increasing MTL, SP, and AchE levels and inhibiting ET, SS, and VIP can effectively improve intestinal function and promote constipation relief [179]. Meanwhile, tryptophan generation has a significant accelerating effect on gastrointestinal motility. In this pathway, L-tryptophan, 5-HTP, and other intermediate metabolites are substrates produced by neurotransmitters and neuromodulators, which can enhance the sensitivity of visceral nerves in the gastrointestinal tract and stimulate gastrointestinal motility. Several studies have shown that NDOs can relieve constipation by activating the 5-HT signaling pathway [179,183]. In addition, it is equally important that bile acids regulate constipation through the metabolism of intestinal bacteria, such as *Bacteroides* [178].

**Figure 4:**
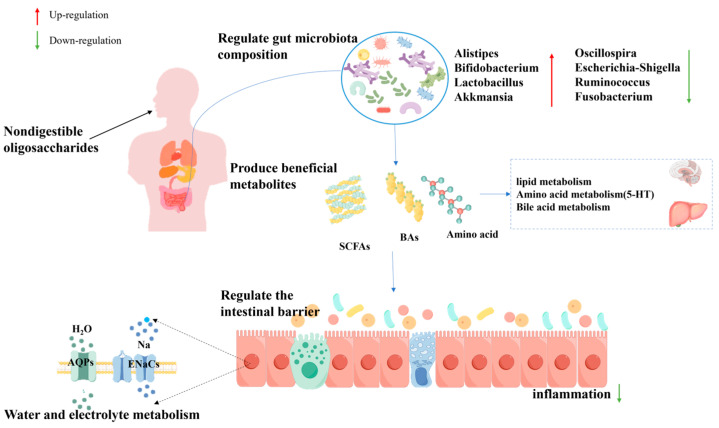
Schematic diagram of the mechanism of action of NDOs in relieving constipation.

In conclusion, NDOs can effectively relieve constipation through a variety of mechanisms, such as promoting intestinal motility, enhancing intestinal barrier function, and regulating immune responses (Figure 4). These findings indicate the potential value of NDOs in the treatment of constipation, further supporting the direction of research to improve constipation by targeting the gut microbiota and its metabolic functions.

## 5. Conclusions and Perspectives

NDOs, as prebiotics, show remarkable potential in alleviating a variety of human chronic diseases, such as diabetes, depression, constipation, colitis, and obesity, by modulating the gut microbiota to produce beneficial metabolites. However, the relationship between the precise structure of oligosaccharides and their fermentation state in the gut remains unclear, which limits their application.

In the future, it is necessary to study the interaction between the structure of NDOs and the gut microbiota, further explore the specific mechanism of NDOs in different chronic diseases, and compare the effects of different oligosaccharide types. The efficacy and safety of NDOs have been demonstrated in multi-center, large-scale human clinical trials. We can make better use of NDOs to develop new dietary supplement and functional foods to provide effective prevention and treatment strategies for chronic diseases.

## Figures and Tables

**Table 1:** Basic information of the main nondigestible oligosaccharides (NDOs).

Name	Glucosidic Linkage	Monosaccharides	DP	Preparation	Natural Sources	References
FOS	β-1,2	Fructose Glucose	2–5	Enzyme degradation, microbial Fermentation	Onions, chicory, wheat	[55,56]
IMO	α-1,3 α-1,4 α-1,6	Glucose	2–5	Enzyme synthesis, enzymatic hydrolysis, enzymatic conversion	Starch, corn, milk, wheat, bran	[57,58]
XOS	β-1,4	Xylose	2–7	Enzymatic conversion	Shoots, corn cob, wheat straw	[59,60]
MOS	α-1,2 α-1,6	Mannose Glucose	2–10	Enzymatic hydrolysis	Carrageenan konjac	[57,61]
GOS	β-1,4 β-1,6	Galactose Glucose	3–6	Enzymatic conversion	Lactose	[55,62]
SOS	α-1,2 α-1,6	Galactose, Glucose Fructose	2–5	Extracted	Soybean	[57]
COS	β-1,4	D-glucosamine	—	Enzymatic hydrolysis	Shell of crustaceans	[63]

## Data Availability

No new data were created or analyzed in this study. Data sharing is not applicable to this article.
